# A Literature Synthesis Indicates Very Low Quality, but Consistent Evidence of Improvements in Function after Surgical Interventions for Primary Osteoarthritis of the Elbow

**DOI:** 10.1155/2013/487615

**Published:** 2013-01-31

**Authors:** Joshua I. Vincent, Anthony A. Vandervoort, Joy C. MacDermid

**Affiliations:** ^1^Faculty of Health Sciences, University of Western Ontario, Elborn College, Room 1424, 1201 Western Road, London, ON, Canada N6G 1H1; ^2^Schools of Physical Therapy and Kinesiology, Faculty of Health Sciences, University of Western Ontario, Room 1400, Elborn College, 1201 Western Road, London, ON, Canada N6G 1H1; ^3^School of Rehabilitation Science, McMaster University, Hamilton, ON, Canada L8S 1C7; ^4^The Hand and Upper Limb Center, St. Joseph's Hospital, 268 Grosvenor Street, London, ON, Canada N6A 4L6

## Abstract

*Background*. Primary osteoarthritis of the elbow is a debilitating disease with an overall incidence of about 2%. Pain and reduced motion (ROM) lead to disability and loss of functional independence. *Purpose*. To critically review the literature on patient-related important functional outcomes (pain, ROMs and functional recovery) after surgery for primary OA of the elbow, utilizing the 2011 OCEBM levels of evidence. *Design*. A literature synthesis. *Results*. Twenty-six articles satisfied the inclusion and exclusion criteria; 25 of the studies were at level IV evidence, and 1 at level III. All three surgical techniques led to improvement in pain, ROM, and functional recovery in the short- and medium-term follow-up. Long-term follow-up results, available only for open joint debridement, showed recurrence of osteoarthritic signs on X-ray with minimal loss of motion. Recently, there seems to be an increased focus on arthroscopic debridement. *Conclusion*. The quality of research addressing surgical interventions is very low, including total elbow arthroplasty (TEA). However, the evidence concurs that open and arthroscopic joint debridement can improve function in patients with moderate-to-severe OA of the elbow. TEA is reserved for treating severe joint destruction, mostly for elderly individuals with low physical demands when other intervention options have failed.

## 1. Introduction

Primary OA of the elbow is a debilitating disease with an overall incidence of around 2% [1]. Men are more commonly affected than women at a ratio of 4 : 1 [2–4]. Primary OA of the elbow has had less focus than lower extremity joint arthritis, but can cause substantial disability. “Elbow arthritis” is an umbrella term, which would include rheumatoid arthritis, haemophilic arthritis, posttraumatic arthritis, septic arthritis, and crystalline arthropathy. Primary OA of the elbow can be difficult to differentiate from posttraumatic arthritis [2], and the reconstructive approaches are similar. The impairments most commonly associated with primary OA are pain, and loss of joint motion, strength, and function. 

In the management of primary OA of the elbow, surgical interventions are used when conservative measures like physiotherapy and medical management fail. The most common indications for surgery are end range pain, stiffness, loose bodies, and locking of the elbow joint. There are a number of surgical options available for the management of primary OA of the elbow. The preferred surgical options depend on the dominant clinical feature and radiographic changes [5]. The earliest evidence in the literature dates back to 1952 when Knight and Van Zandt [6] reported the results of arthroplasty with resection of the articular surface. Then came reports of other techniques such as the Outerbridge-Kashiwagi procedure [3], arthroscopic debridement [7], interposition arthroplasty [8], ulnohumeral arthroplasty [9], and total elbow arthroplasty. Technical details of these surgeries are beyond the scope of this paper since the focus of this paper is the functional outcomes that result. A basic description of the commonly used surgical procedures is described later.

 Open joint debridement: open joint debridement is considered as a “house cleaning” procedure that is a traditional choice for primary osteoarthritis of the elbow [34]. This procedure involves removal of impinging osteophytes, capsular release, joint debridement, and loose body removal. Outerbridge was a pioneer in this procedure, and later in 1978 Kashiwagi reported success with this procedure [3]. Later this procedure was called the “Outerbridge-Kashiwagi arthroplasty” (O-K arthroplasty) [35]. A modification of this procedure that was based on site of exposure, trephine debridement, and excision of distal humerus along with the olecranon and the coronoid tips was termed as ulnohumeral arthroplasty (UHA) [9]. Another method of open debridement is called the “column procedure,” wherein UHA is combined with anterior debridement and capsular release. In this paper these types of surgeries are classified under one common umbrella term known as open joint debridement.

 Arthroscopic debridement: arthroscopic debridement is indicated in cases of primary OA of elbow presenting with loose bodies or modest osteophyte formation with impingement pain at the extremes of motion but not in the midarc [5]. It is most commonly used in the case of younger individuals, who have less extensive joint disease. This procedure involves removal of impinging osteophytes, capsular release, joint debridement, and loose body removal. Complete debridement may not be possible. Advantages of this procedure are less intraoperative bleeding decreased postoperative pain resulting in early recovery [36]. However, since it requires specialized equipment and expertise, this procedure may not be available in all situations. 

Total elbow arthroplasty: in primary osteoarthritis of the elbow, total elbow arthroplasty (TEA) is reserved for the elderly since there is a concern about the length of time that the joint would survive. It involves replacement of both joint surfaces and thus is indicated for extensive joint destruction, in people who have minimal physical demands and in whom other means of management have failed. TEA prostheses are classified into linked and unlinked prostheses. These names are used interchangeably with semiconstrained and unconstrained prostheses, respectively [37].

Pain and loss of the elbow range of motion have been reported to be of major concern for patients with primary OA of the elbow. The presence of these can affect upper extremity function since the elbow is critical to where the hand is positioned in space. Therefore, pain relief, improvement in range of motion (ROM), and good functional recovery are the outcomes expected from surgical procedures for primary OA. To date, there is no report in the literature that summarizes these three patient important outcomes after surgery in individuals with primary OA of the elbow.

The 2011 OCEBM levels of evidence [38] developed by the center for Evidence Based Medicine were designed to help clinicians and researchers make informed decisions about the quality of clinical evidence. The literature on the outcomes after surgical management of primary OA has not been classified based on the strength of evidence. Thus, there is a need to determine the quality and content of evidence regarding these surgical procedures. Determining “best evidence” for these surgical procedures would help clinicians and researchers to make informed decisions in patient care and designing clinical trials [39].

Hence the purposes of this review are to

- adjudge the methodological quality and classify the available evidence on the outcomes after surgical management of primary OA of the elbow using the 2011 OCEBM Levels of Evidence table,
- summarize and critically review the available evidence in the literature on expected patient important functional outcomes (pain, ROM, and functional recovery) after surgery in older adults with primary OA of the elbow.

## 2. Methodology

**Figure 1:**
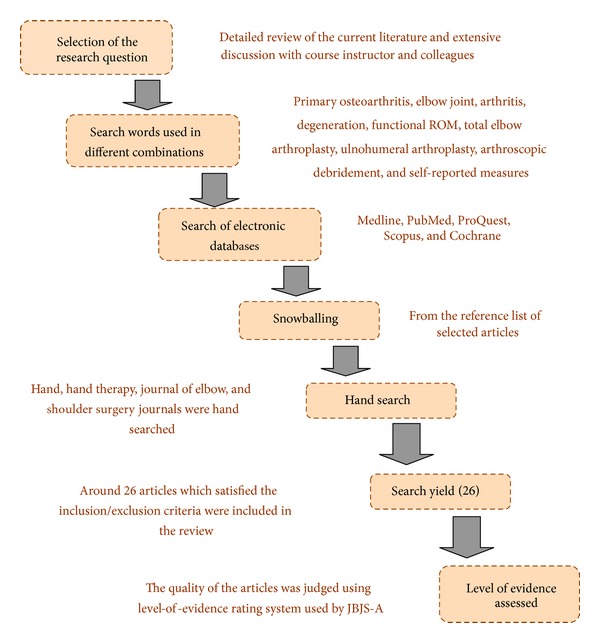
Flow chart showing the methodology of the review.

Electronic databases Medline (1970–2012), CINAHL (1982–2012), Proquest (1982–2012), and Scopus (Inception-2012) were searched-for articles. The following search terms were used in different combinations: “Primary Osteoarthritis,” “Elbow joint,” “Elbow arthritis,” “Degeneration,” “Age-related changes,” “Articular changes,” “Total elbow arthroplasty,” “Ulnohumeral arthroplasty,” “Arthroscopic debridement,” “Functional range of motion,” “Self-reported measures,” and “Functional recovery.” “Snowballing,” that is, the reference lists of the selected articles was also screened to find any missing articles (see Figure 1).

The following inclusion criteria were used:

- surgical management of primary osteoarthritis of elbow,
- age group—any,
- level of evidence—any,
- outcome reported should include at least one of the patient important outcomes (pain, ROM, and functional recovery),
- articles published in English language.

The following exclusion criteria were used:

- management of other types of elbow arthritis like rheumatoid arthritis, posttraumatic arthritis, or haemophilic arthritis.

Once the articles were selected, the full text was retrieved and they were classified using the 2011 OCEBM Levels of Evidence. Then information on the expected patient important outcomes of pain, range of motion, and functional recovery was extracted from these papers using a structured form. A descriptive synthesis of findings was done. Under the range of motion we focussed on the flexion-extension arc of the elbow. Supination-pronation ROM was not summarized, as with the majority of cases with primary OA of the elbow, forearm rotation is minimally affected due to minimal involvement of the radiohumeral joint [5], and it was rarely addressed in published studies. Information on patient satisfaction was also extracted.

## 3. Results

### 3.1. Search Results

Overall, 28 articles that satisfied the inclusion and exclusion criteria were retrieved and reviewed. Of these, two were not included because they had a mix of patients with other elbow disorders and did not report results for primary OA patients separately. Thus, 26 articles were retained. Of the 26 articles 15 were on open joint debridement; 7 were on arthroscopic joint debridement; 3 were on total elbow arthroplasty; and 1 was a retrospective comparative study comparing the effectiveness of open with arthroscopic joint debridement. 

### 3.2. Levels of Evidence

The methodological quality and level of evidence were assessed. In terms of level of evidence, out of the 26 studies that satisfied the inclusion criteria, none of the studies were of the level I or level II. One study was a nonrandomized control trial (level III), and the rest of the studies were either case series or were retrospective case reviews (level IV). 

### 3.3. Open Joint Debridement

#### 3.3.1. Change in Pain

All the studies reported good to excellent pain relief after open joint debridement (see Tables 1, 2, and 3). However, studies used different measures to evaluate pain. Two studies have used visual analog scale (VAS) [10, 12]; 3 studies [9, 16, 19] have used the pain grading system proposed by Morrey which is a 0–3 Likert scale. Two studies [21, 22] have used nonvalidated Likert scales; 2 studies [13, 20] have used pain subscales of elbow scoring systems. In spite of reporting good pain relief, 7 studies did not report the outcome measure they used [11, 14, 15, 17, 18, 23, 24].

#### 3.3.2. Change in ROM

The reports on the improvement in ROM after open joint debridement studies have given variable results (see Tables 1, 2, and 3). The average increase in ROM across the studies was 24 degrees (range 16–49.3). Ugurlu et al. [10] in their retrospective case study reported the highest increase in the flexion-extension arc of the elbow, a mean increase of 49.3 degrees after following 10 patients up for 18 months. The lowest change in mean ROM was 16 degrees reported by Tashjian et al. [12] after following 18 elbows for 85 months. 

The earliest report of success in this particular subset of the population was reported by Morrey (1992) [9]. Morrey performed UHA on 15 elbows and reported good pain relief and a mean increase of 21 degrees in the elbow flexion-extension arc after following them up for 33 months. A couple of years later, Tsuge and Mizuseki (1994) [24] reported an increase of 34 degrees after following 29 elbows managed with UHA over 5 years on average. 

Cohen et al. (2000) [20] compared open joint debridement to arthroscopic debridement in 44 elbows. After following them up for nearly three years, they found that open debridement had a better mean increase in flexion-extension arc of 21 degrees, compared to a mean increase of 7 degrees with arthroscopic debridement. Sarris and associates (2004) [16] in a series of 44 elbows managed with UHA reported a mean increase of 32 degrees in the flexion-extension arc at 3-year followup. The latest report by Rettig and colleagues (2008) [11] showed a mean increase of 30 degrees in 21 elbows after following them for more than 5 years on average after UHA. Oka (2000) [21] published the results of 50 elbows treated with UHA followed up for nearly 5 years. He reported a mean increase of 24 degrees in the flexion-extension arc of the elbow.

#### 3.3.3. Change in Function

All the studies reported acceptable levels of functional restoration. Change in function was assessed using different measures (see Tables 1, 2, and 3). The Japanese Orthopedic Association elbow scoring system was used by Wada et al. [13] and Tsuge and Mizuseki [24] who reported a mean increase of 23 and 29.3 points, respectively. Most of the studies [11, 12, 14, 17, 18, 20] used the Mayo elbow performance index (MEPI) to assess functional recovery. All of these studies reported good to excellent functional recovery. However Tashjian et al. [12] have found that the MEPI did not correlate well with Disabilities of the Arm Shoulder and Hand (DASH) (a self-report measure) questioning the validity of MEPI's scores. One study [10] used Andrews and Carson's (A-C) scoring system finding a 90% improvement in function (before 88.5; after 168.5). Only 2 studies [12, 17] have used the DASH, a region-specific self-reported questionnaire.

#### 3.3.4. Complications and Recurrence of OA

Some of the complications reported were instability and pain at rest [11], ectopic bone formation [24], ulnar neuropathy [9], and cubital tunnel syndrome [23]. Cohen et al. [20] reported 1 patient who required revision at 2-year followup due to poor results. In the studies with long-term followup, there was a substantial loss of the gained flexion-extension arc and recurrence of osteophytes. Wada et al. [13] reported the average loss of extension increased from 19 degrees in the first year followup to 26 degrees at the latest followup, which translates to a loss of 7 degrees in 33 elbows followed for up to 10 years on an average after open joint debridement. Secondly, a tendency of advancing roentgenographic osteoarthritic changes was reported by Minami and associates (1996) [23] in most of the cases in their case series followed for 8 to 16 years after O-K arthroplasty. Oka [21] has also found recurrence of osteophytes and loss of ROM in long-term followup. 

### 3.4. Arthroscopic Debridement

#### 3.4.1. Change in Pain

All the studies reported excellent pain relief after arthroscopic debridement (see Tables 4 and 5).  Three of the studies [25, 27, 28] have used the VAS and have reported dramatic changes in pain levels. Krishnan et al. [27] after following 11 elbows for 26 months reported a change in the mean VAS scores from 9.2 (before) to 1.43 (final followup). Redden and Stanley [31] used a linear analog scale and reported good pain relief but with insignificant ROM changes. Others [20, 30] have adopted pain subscale scores of elbow scoring systems like MEPI and a scoring system developed by Morrey and have reported good improvement in pain levels. One study [26] used a Likert scale from 0 to 5 and another study [29] did not describe their pain measure, but noted that there was good pain relief.

#### 3.4.2. Change in ROM

The reports on ROM changes are variable (see Tables 4 and 5). The earliest report was given by Redden and Stanley (1993) [31]. They reported excellent pain relief, a high level of patient satisfaction, but insignificant increase in the flexion-extension arc, in a small study of 12 elbows after a mean followup of 16 months. Cohen et al. (2000) [20] retrospectively compared open versus arthroscopic debridement in 44 elbows followed up for 35.5 months. Of the 44 elbows 26 were managed by arthroscopic debridement. They reported good pain relief, but a smaller mean increase in the flexion-extension arc of 7 degrees. 

While some of the earlier studies reported suboptimal outcomes, there is a trend for better functional outcomes over time. After following 30 subjects for 42.5 months, Kim and Shin (2000) [29] reported excellent pain relief and a substantial increase in flexion-extension arc of 36 degrees. In a series of 41 cases (42 elbows) Adams and Steinmann (2008) [26] reported an increase of 27.3 degrees in the flexion-extension arc after a mean followup of 44 months. Krishnan et al. (2007) [27] in their case series involving 11 elbows found a large improvement in the flexion-extension arc (73 degrees) in a 26-month followup. Kelly et al. [28] reported a mean increase of 21 degrees in 25 elbows followed for more than 5 years. Most recently Degreef et al. (2010) [25] performed the Outerbridge-Kashiwagi procedure arthroscopically in a series of 19 cases (20 elbows) and followed them up for 24 months on average and reported a mean increase of 29 degrees in the flexion-extension arc and excellent pain relief. 

#### 3.4.3. Change in Function

Four studies [20, 25–27] reported function using the MEPI and have noted good to excellent results (MEPI score > 75) in the majority of the cases. Kelly et al. [28] have used both subjective and objective parts of the A-C scoring system for elbow and reported dramatic changes in levels of function. One study [30] used the elbow classification system developed by Morrey and reported better function. Notably none of these studies other than Kelly et al. [28] have used self-report measures to measure functional outcomes.

#### 3.4.4. Complications and Recurrence of OA

Unlike the open debridement studies, complications were quite minimal. Kim and Shin [29] have reported on a case of transient median nerve palsy “occurred immediately after the operation.” Adams and Steinmann [26] observed 2 complications—one was a case of heterotrophic ossification and the other was ulnar nerve dysesthesia—in their retrospective review of 42 elbows. Recurrence has not been reported in any of the reviewed papers.

### 3.5. Total Elbow Arthroplasty

#### 3.5.1. Change in Pain

All three studies addressing TEA for primary OA have reported excellent pain relief. Naqui et al. [32] reported of a substantial pain relief with the VAS scores dropping from 8 preoperatively to 0 at the final followup at 57.6 months. Kozak et al. [1] also reported excellent pain relief with pain being 3/3 preoperatively to being 0.4/3 during final followup. Espag et al. [33] also have reports of mild or no pain in 10 cases out of the 11 elbows reconstructed.

#### 3.5.2. Change in ROM

The evidence is limited, but suggests TEA improves ROM (see Table 6). After following 11 cases for an average of 57.6 months, Naqui et al. (2010) [32] reported excellent pain relief and a substantial increase in the mean flexion-extension arc by 40 degrees. Similar results were reported by Kozak et al. (1998) [1] and Espag et al. (2003) [33]. Kozak et al. [1] have reported a mean increase in the flexion-extension arc of 34 degrees after 63 months of mean followup while Espag and his associates [33] reported it as 38 degrees after 68 months of followup. 

#### 3.5.3. Change in Function

Of the three studies identified, two reported on using scoring systems to assess functional recovery. Kozak et al. [1] used MEPI (surgeon administered measure) and reported a preoperative score range from 15 to 40 and an excellent followup score ranging from 80 to 100. Naqui et al. [32] have also used MEPI and reported that 9 out of 11 cases had good to excellent results at the final followup; they also used American Shoulder and Elbow Surgeons elbow form (ASES-e) and reported a drastic improvement from 2/36 at preoperatively to 33/36 at final followup.

#### 3.5.4. Complications and Revisions

All 3 TEA studies have reported some complications. Naqui et al. [32] have reported one postoperative transient ulnar neuropathy, which resolved and one intraoperative medial condylar fracture. Espag et al. [33] have also reported two ulnar neuropraxias, which resolved, and 2 superficial wound infections. Kozak et al. [1] reported complications in 4 out of 5 patients which included subluxation, fracture of a humeral component with particulate synovitis, heterotopic ossification, recurrent osteophyte formation, and transient ulnar neuropathy. They were required to make revisions in two cases. 

## 4. Discussion

The overall quality of evidence found addressing elbow surgeries in a primary OA population was very low. A formal randomized clinical trial is yet to be done, and so the present strength of evidence consisted of level III and level IV studies. However, nearly all the studies reported excellent pain relief, substantial gain in ROM, and functional recovery. In reconstructive procedures pre-post testing provides important evidence since it expected outcomes. However, a lack of trials limits the ability to compare different treatment options. Clinicians should be aware that best evidence supports surgical intervention, but does not help select optimal timing or a specific procedure.

The evidence is lacking on comparative outcomes. Some studies addressing total elbow arthroplasty for other types of arthritis such as inflammatory or posttraumatic arthritis may not provide outcomes that are similar to those seen in osteoarthritis. However, reviews that show these procedures working across other forms of arthritis could increase our confidence that joint debridement or reconstructive procedures are safe and effective [40, 41]. Most notably there is a lack of RCTs comparing different surgical options either in terms of short- or long-term benefit. Some studies had to be excluded from this paper because it was not clear which types of arthritis were included in their sample. As the amount of studies increases it will be possible to use meta-analysis to determine if the subgroups of arthritis have different outcomes across different procedures. At present we cannot compare outcomes across procedures since all the articles, except one, were case series or retrospective case studies (level IV). 

In this paper we found that there were multiple studies (*n* = 16) reporting on the effectiveness of open joint debridement, and these had the longest followups reported. All studies found excellent initial pain relief, gain in ROM, and functional recovery. However the studies with longer followups indicated recurrence of OA symptoms. Minami et al. (1996) [23] indicated a tendency of advancing roentgenological changes in most of the elbows in their case series 10 years after O-K arthroplasty. Oka et al. (1998) [22] observed OA changes in a few of their patients after 5 years of followup. These results suggest that this procedure can provide predictable short-term relief but cannot guarantee a lack of progression of OA. Further investigations are recommended to gain better understanding about the mechanism of recurrence of primary OA of the elbow, the biological processes involved in it, and how this could be prevented through conservative and/or surgical procedures.

**Figure 2:**
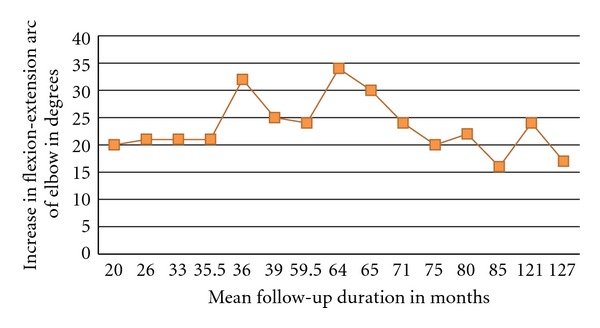
Graph showing trends in the flexion-extension arc as mean duration of followup increases for open debridement.

When the mean duration of followup for the studies on open joint debridement was plotted against the mean increase in range of flexion-extension arc, an interesting trend was noted in that the increase in flexion-extension arc was slow in the initial years, and it gradually increased during the middle of the timeframe with the highest gain in range of motion between the 3rd and 8th years of followup and then started to taper as the duration of followup increased (see Figure 2). This trend reported across studies has also been observed within individual longitudinal studies [13]. This may suggest that motion continues to improve for a period after the acute recovery period possibly due to increased functional use, but that with aging and possible progression of the disease function will again deteriorate. Longer and larger prospective cohort studies are needed to further understand how aging, joint degeneration progression and other factors affect upper extremity function. Similar graphs were not plotted for the outcomes of arthroscopic debridement and total elbow arthroplasty because they had very few studies supporting their efficacy, and the average followup duration was also low.

The trend in early studies of arthroscopic debridement had far less promising results [20, 31]. However, later studies have shown a substantial gain in ROM [25, 27, 29]. It is expected that when a surgery is first introduced, it should demonstrate benefit; however the nature of this improvement may change as the procedure is transferred more widely into practice. If the initial studies are performed in “ideal candidates,” then the outcome may become poorer as the indications are extended to broader indications. Conversely, if the procedures can be improved by progressive innovations in surgical or postoperative techniques, including rehabilitation, then outcomes might improve over time. The latter appears to be the case for arthroscopic debridement. Temporal effects can affect different procedures to a different extent. For example, joint arthroplasty outcomes are expected to improve over time as the implant design improves. Hence, comparative differences between procedures may change over time as well. Variations across case series are expected as these would reflect the nature of the surgical populations, surgeons, settings, and procedures across different contexts. In the absence of comparative trials where outcome are assessed concurrently, we had to look for temporal cohort effects as a low quality means of assessing how outcomes are changing with different procedures 

The best surgical intervention for the treatment for symptomatic elbow osteoarthritis is controversial. This paper could not provide any strong recommendations in support of the use of one surgical procedure over the other as all the studies were of low quality. However, from the information we retrieved, the open and arthroscopic debridement surgeries seem to have a definite role in management of elbow OA. Both have excellent pain relief and provide gains in ROM and functional recovery in the short term. Open debridement reports have shown that there were recurrences in symptoms of OA in the long term. Open debridement has the potential advantage that it provides full visualization and allows for treatment of all compartments of the elbow. However, there is an extensive soft tissue dissection and division of at least one collateral ligament. This may lead to instability and stiffness. Arthroscopic debridement allows access to the whole joint with very minimal dissection. However, this procedure requires expertise and specialized equipment. Arthroscopic procedures have many advantages in diagnostics and treatment, including less scarring, complete access to the joint cavity, fewer complications, shorter recovery period, and cosmetic advantages. Thus, the use of the procedure is likely to increase over time, although long-term followup studies are needed. There was only one retrospective comparative study [20] that compared open versus arthroscopic debridement and it found open debridement to be more effective in terms of range of motion. Further randomized clinical trials are required in this area to compare the effectiveness of these two procedures.

The total elbow arthroplasty (TEA) is a more extensive reconstruction and complications are a concern; including the potential for infections, triceps paralysis, prosthetic loosening, and prosthetic failure. However, complication rates are decreasing over time. The limited evidence suggests good results in patients with primary OA, but long-term survivorship should be studied. Further, differences between linked versus unlinked prostheses need to be investigated in high quality studies. 

This study focused on functional outcomes and noted considerable differences across studies in how these were measured. Clinician-based measures (CBO) often provide a composite score that summarizes a number of aspects of impairment and function. The MEPI and JOA were CBOs reported in this paper. Patient-reported outcome measures (PRO) should be considered an important benchmark and are now required by the FDA to approve new implants. Of the studies included in this paper only 16% used a PRO. The DASH, PREE, and ASES-e are all PROs that can be used to evaluate outcomes of elbow surgery [42]. The current trend in orthopaedic research is an increased use of patient self-reported measures of function, although clinician-derived outcome scores are still common [43]. One of the studies included in this paper has found results suggesting “a discrepancy between patient-derived functional outcome measures and physician-derived scoring systems in patients after UHA” [12]. This clearly questions the validity of the scores obtained through surgeon-rated elbow scoring systems since most were not developed using appropriate clinical measurement procedures. However, it is more likely that PROs measure different aspects of outcome than CBOs and that both are needed for a more comprehensive view of the outcomes achieved.

Patient satisfaction is a controversial outcome when used as an indicator of surgical outcomes. In the shoulder, it has been shown not to relate either to PRO of function or physical impairments or expert assessment [44]. Nevertheless, in elective procedures one assumes that patient satisfaction is an important dimension to consider. In the current paper, we found that many of the studies did not assess patient satisfaction. When used, either a simple Likert scale or a 0–10 VAS scale was selected. If measured with a single item, then we recommend the use of VAS or a 0–10 scale as it has the ability to capture a greater variability in patient responses than a Likert scale. Another important outcome that was neglected in the studies we retrieved was quality of life. Only 1 of the 26 articles assessed quality of life after surgery [12].

Our study had limitations. Our primary limitation was with respect to the evidence itself. Current best evidence is of very low quality, and prevents any strong recommendations. In addition, we included studies only in English. It is possible; but unlikely, that we missed high quality studies published in other languages. We excluded few studies as they had a mixed sample of various elbow disorders, and the authors of those studies did not report outcomes for primary OA cases separately. This might have cost us valuable information on the efficacy of these surgical procedures. However, we choose this path rather than including undefined samples. Finally, we could not always extract useful information because of improper reporting of results. Some studies failed to cite the measure or specific data used to assess pain. 

All the conclusions made in this paper are suggestions based on the available literature which is limited at source in terms of its quality. We did not use quantitative quality appraisals or quantitative meta-analysis because the literature was insufficient for these methods. Available studies could not provide robust estimates that would inform clinical practice. We used a narrative literature synthesis approach to summarize the literature, and quantitatively analyzed longitudinal trends across studies where possible [45, 46]. These general time trend across studies can inform how practice outcomes change over time which can be due to both changes in indications and changes in surgical techniques/implants. 

## 5. Conclusion

This critical review provides very preliminary support for the use of open joint debridement, arthroscopic debridement and total elbow arthroplasty to improve pain, joint motion and elbow function. Arthroscopic debridement seems to be a viable option where available since it is the least invasive and has good surgical outcomes. Open joint debridement may be preferred by some surgeons since there are more studies supporting its efficacy. Given the lack of comparative studies surgeons experience and preference may be used to select the optimal procedure. Total elbow arthroplasty is reserved for the elderly with minimal physical demands and is effective in improving pain, ROM, and function when other techniques become inappropriate. There is a pressing need for high quality multicentered randomized control trials with longer duration of followup using valid patient self-reported measures.

## Figures and Tables

**Table 1:** Summary table for outcomes of open joint debridement-1.

Study and year	Design	Level of evidence	*n*	Followup (in months)	Pain Δ	ROM Δ	Function Δ
Ugurlu et al., 2009 [10]	Retrospective case study	IV	10	18	VAS (before 8; after 3.1)	49.3	Andrews and Carson scoring (before 88.5 to after 168.5)
Rettig et al., 2008 [11]	Case series	IV	18 (21 elbows)	65	Good improvement in pain. No measure described	30	MEPI (after 85.8)
Tashjian et al., 2006 [12]	Case series	IV	17 (18 elbows)	85	VAS	16	(i) Hospital for special surgery elbow score after 70 (9 good to excellent results) (ii) MEPI after 83 (15 good to excellent results) (iii) DASH after 9.75 (iv) VAS Function 7.9 after (v) SF-36
Wada et al., 2004 [13]	Case series	IV	32 (33 elbows)	121	before 13 improved to after 27	24	JOA elbow scoring system (before 60 to after 83)
Vingerhoeds et al., 2004 [14]	Case series	IV	15 (16 elbows)	20	Good pain relief. No measure reported	20	MEPI (before 63 to after 88)

**Table 2:** Summary table for outcomes of open joint debridement-2.

Study and year	Design	Level of evidence	*n*	Followup (months)	Pain Δ	ROM Δ	Function Δ
Allen et al., 2004 [15]	Case series	IV	9	26	Good pain relief. No measure reported	21	—
Sarris et al., 2004 [16]	Case series	IV	15	36	Morrey's system (0–3 Likert scale). All patients reported 0 after-operatively	32	No measure used. Reported that all patients returned to work
Phillips et al., 2003 [17]	Case series	IV	20	75	Good pain relief. No measure reported	20	(i) DASH (17 with good or excellent results) (ii) MEPI (13 with good or excellent results) (iii) 12 out of 16 working before-operatively returned to work
Antuña et al., 2002 [18]	Case series	IV	45 (46 elbows)	80	76% had complete pain relief. No measure reported	22	MEPI (before 55 to After 83)
Forster et al., 2001 [19]	Case series	IV	43 (44 elbows)	39	Morrey's system (0–3 Likert scale). (1.8 before to 1.1 after)	25	—

**Table 3:** Summary table for outcomes of open joint debridement-3.

Study and year	Design	Level of evidence	*n*	Followup (months)	Pain Δ	ROM Δ	Function Δ
Cohen et al., 2000 [20]	Nonrandomized control study	III	18 out of 44	35.5	0–6 Likert scale from MEPI (after 2 points)	21	MEPI
Oka., 2000 [21]	Case series	IV	50	59.5	0–3 grading scale (before 2.46 to after 0.42)	24	—
Oka et al., 1998 [22]	Case series	IV	36 (38 elbows)	71	0–2 grading scale (before 2 to after 0.24)	24	—
Minami et al., 1996 [23]	Case series	IV	44	127	27 out of 44 reported good pain relief. No measure reported	17	—
Tsuge and Mizuseki, 1994 [24]	Case series	IV	28 (29 elbows)	64	Good pain relief. No measure reported	34	(i) JOA elbow scoring system (before 55.2 to after 84.5) (ii) Most of the patients returned to work
Morrey., 1992 [9]	Case series	IV	15	33	Morrey's system (0–3 Likert scale). (2.06 before to 0.33 after)	21	Morrey's elbow scoring system (before 51 to after 75)

**Table 4:** Summary table for outcomes of arthroscopic joint debridement-1.

Study and year	Design	Level of evidence	*n*	Followup (months)	Pain Δ	ROM Δ	Function Δ
Degreef et al., 2010 [25]	Retrospective review	IV	19 (20 elbows)	24	VAS (before 5.8 to after 1.8)	29	(i) MEPI (before 54 to after 88; good to excellent results in 16 elbows)
Adams and Steinmann, 2008 [26]	Retrospective review	IV	41 (42 elbows)	44	0–5 Likert scale. (2.86 before to 1.43 after)	27.3	(i) MEPI (before 67.5 to after 84.4; good to excellent results in 81% of elbows)
Krishnan et al., 2007 [27]	Case series	IV	11	26	VAS (before 9.2 to after 1.7)	73	MEPI (before 58 to after 89; good to excellent results in 11 elbows)
Kelly et al., 2007 [28]	Case series	IV	24 (25 elbows)	67	VAS (before 7 to after 2)	21	Andrews and Carson scoring (subjective scoring—before 45 to after 82; objective scoring—before 69 to after 93)
Kim and Shin., 2000 [29]	Case series	IV	30	42.5	27 patients reported good pain relief. Measure not reported	36	—

**Table 5:** Summary table for outcomes of arthroscopic joint debridement-2.

Study and year	Design	Level of evidence	*n*	Followup (months)	Pain Δ	ROM Δ	Function Δ
Cohen et al., 2000 [20]	Nonrandomized control study	III	26 out of 44	35.5	0–6 Likert scale from MEPI (after 2.9 points)	7	MEPI
Ogilvie-Harris et al., 1995 [30]	Case series	IV	25	35	30-point scale from Morrey's scoring system for elbow (improved from before 19 to after 28)	—	(i) 100 point elbow classification system developed by Morrey (ii) 14 patients returned to work or full activity
Redden and Stanley., 1993 [31]	Case series	IV	12	16	Linear analog scale (good pain relief)	Insignificant change in ROM	—

**Table 6:** Summary table for outcomes of Total elbow arthroplasty.

Study and year	Design	Level of evidence	*n*	Followup (months)	Pain Δ	ROM Δ	Function Δ
Naqui et al., 2010 [32]	Retrospective case review	IV	11	57.6	VAS (before 8 to after 0)	40	(i) ASES (improved from before 2/36 to after 33/36) (ii) MEPI (9 good to excellent and 2 fair results)
Espag et al., 2003 [33]	Retrospective case review	IV	11	68	Mild or no pain in 10 patients. No measure reported	38	—
Kozak et al., 1998 [1]	Case report	IV	5	63	(0–3 likert scale). (3 before to 0.4 after)	34	MEPI (before range 15–40 to after range 80–100)
